# Sailing together: challenges for an inclusive team of visually impaired and sighted athletes at Kiel Week

**DOI:** 10.3389/fspor.2025.1602925

**Published:** 2025-06-13

**Authors:** Steffen Greve, Frederik Bükers, Paula Bodenstedt, Katrin Steinvoord, Claus Krieger

**Affiliations:** ^1^ Department of Teaching Competence in Sport, Institute for Sports Science, Humboldt-University of Berlin, Berlin, Germany; ^2^ Unit for Physical Education and Sports Didactics, Institute for Physical Activity, Sports and Health, Leuphana University Lüneburg, Lüneburg, Germany; ^3^ Subject Group Movement, Games and Sports, Department of Education, University of Hamburg, Hamburg, Germany

**Keywords:** competitive sports, inclusion, adaptive sailing, communication, utilization-focused evaluation

## Abstract

The BAT-Sailing project is a joint project of the Norddeutscher Regatta Verein and FC St. Pauli Segeln, which enables people with and without disabilities to pursue sailing together in a performance-oriented manner as part of training and regattas. The project originally started with the intention of realizing the joint sailing of sighted and blind athletes. This article presents the scientific monitoring that was carried out at the request of the BAT Sailing Team in order to scrutinize and optimize the practice of the BAT Sailing Team [The name is derived from the word “bat” and alludes the symbolic transfer: cannot see (well) but can fly (or sail)]. The evaluation was carried out according to the Patton approach of a utilization-focused evaluation, which places the needs and values of the users at the center of the evaluation. The evaluation took place over three consecutive years (2021, 2022, 2023) and included interviews with the athletes, coaches and organizers of the BAT Sailing Team. The results showed that communication between sighted and blind athletes plays a special role and that the athletes learn to understand and support each other. Within the process of the evaluation it was able to identify communication as a strength that has developed in the joint sailing of people with and without visual impairments and that benefits above all people without disabilities. The results of the evaluation show that the utilization-focused evaluation is an effective tool for improving the practice of an inclusive sailing team that wants to act in a performance-oriented manner but also wants to ensure the participation of all potentially interested parties, regardless of their dis/abilities. The results of the evaluation can also be transferred to other inclusive sports projects that face a similar challenge.

## 1 Introduction

The inclusive sailing initiative BAT-Sailing started as a joint project between the Norddeutscher Regatta Verein and FC Sankt Pauli Segeln and is now primarily organized by the association Wir sind wir - Inclusion in Sailing. What is special about this project is that people with and without disabilities pursue sailing together in a performance-oriented manner as part of training and regattas. It is primarily about sighted and blind people sailing together in the J/70 boat class. The J/70 is a planing keelboat officially recognized as a one-design class by the International Sailing Federation. It is typically raced in regattas with a crew of approximately four sailors. To date, there are only a few inclusive sailing sports on offer in Germany, and most of these have no explicit competitive character or deliberately negate this (1).

The research project IncluSail (Inclusion in and through sailing) is conducting scientific research to accompany BAT-Sailing. This evaluation research, initiated at the request of the BAT Sailing stakeholders, critically scrutinizes their approach in the context of the established and (previously) exclusive sailing event Kiel Week. Kiel Week is an internationally renowned sailing regatta held annually in Kiel, Germany. Recognized as one of the largest sailing events worldwide, it features competitions across 16 national and international boat classes, as well as all ten Olympic sailing classes. Each year, the event attracts approximately 5,000 sailors representing more than 50 countries, competing with around 2,000 boats. Those responsible of the BAT Sailing team wanted to question and optimize the actions within the team with professional scientific support, preferably in direct exchange. After examining possible alternative methods, the approach of a utilization-focused evaluation according to Patton (2, 3) was selected as suitable for this purpose and has been pursued since the start of the accompanying research in 2021. This article presents in particular the research process within the framework of the utilization-focused evaluation according to Patton and reports and reflects on the achievement of key results, which lie in the area of communication between the sailors. For this purpose, the central steps, analysis loops and exchange processes are described and shown with the help of the Utilization-Focused Evaluation Checklist according to Patton (4). In a subsequent section, the genesis of the knowledge process in relation to a selected topic area, namely that of joint communication in sailing practice, is examined in more detail as an example. Finally, a discussion is drawn with regard to the potential and limitations of the research methodological approach within the framework of the utilization-focused evaluation.

### 1.1 Sailing for people with disabilities – outline of the initial situation

Sailing has a relatively long tradition in sports practice as a sport for people with disabilities, which is attributed to the fact that participation in sailing can be ensured with relatively little physical effort and technical adaptations can be made to the sailing boat to meet individual requirements (5, 6). However, sailing received particular attention as an official Paralympic discipline between 2000 and 2016. As early as 1996, sailing competitions were held at the Paralympics for demonstration purposes, but were not officially listed and scored. In 2000 and 2004, the 2.4mR (single crew) and Sonar (triple crew) boat classes took part in the competitions. In 2008, the Skud18 (crew of two) was added. All three boats are keelboats, which guarantee a greater or lesser degree of stability in the water (7). This way, adaptive sailing offers opportunities for participation for people with various disabilities. Essentially, this is generated by the fact that individual adaptations to the boat are possible rather than insisting on standardized and therefore potentially disabling norms in the equipment. The adaptation options primarily concern seating and support systems, communication systems and modifications to the boat itself, which can change the handling of the sails and steering (8).

Although the practice of adaptive sailing is highly diverse, international scientific engagement with this topic has so far been limited (9). This must also be noted for paralympic or competitive and regatta-oriented sailing, for which Prokopowicz et al. (10) state that joint and competitive sailing in particular provides an incentive for already active athletes with disabilities to practice sailing. In the last decade, however, there have been studies from various scientific fields, most of which have moved away from competitive sailing. One relatively strong strand of research focuses, for example, on the therapeutic or rehabilitative potential of adaptive sailing for people with disabilities (9, 11). In this regard, MacLachlan (12) state that sailing offers have recently been increasingly used as an intervention in the field of rehabilitative therapy measures for people with disabilities, partly because outdoor offers in this area are in greater demand. Isolated studies in this area outline various potentials for the development or rehabilitation of the mental and physical health and social skills of participants with disabilities (13, 14) or people with mental health problems (15). Such positive effects of adaptive sailing offers are also emphasized for therapeutic measures for children with disabilities (16, 17), as well as, apart from therapeutic measures, for recreational and joint sailing for adults with and without disabilities (18) and explicitly for people with tetraplegia (19). Sailing together by individuals with and without visual impairments is not only seen as a form of inclusive sports participation, but in some cases also explicitly recognized for its enhanced rehabilitative potential. According to Shumova et al. (20), such integrated sailing experiences can foster physical, psychological, and social benefits, including improved orientation skills, greater self-confidence, and a strengthened sense of community and mutual support among participants.

All of the studies outlined here differ from one another to a greater or lesser extent – this applies to core questions, target groups, sample size and research methodology, among other things (9). As a result, the positive effects and potential attributed to adaptive sailing must certainly be viewed critically and put into perspective. However, one thing the contributions have in common is that almost all of them implicitly or explicitly (13, 19) refer to the importance of the accessibility and usability of the boat used (21). This fact also suggests that previous research has placed a clear focus on people with physical disabilities and their participation in sailing. Likewise, less attention has been paid to the practical phenomena of inclusive sailing, i.e., people with and without disabilities sailing together. The latter also applies to the rare research studies that explicitly consider the participation of blind people and people with visual impairments in sailing. Exemplary exceptions from the field of recreational touring sailing explicitly present results that suggest that both the material characteristics of the boat and teamwork, explicitly communication between sighted and blind or visually impaired athletes, harbor potential barriers (14, 22).

It should be noted that the field of competition-oriented or competitive and at the same time inclusive sailing represents a research desideratum and this also applies in particular to the constellation of joint regatta sailing by sighted and blind or visually impaired athletes, which is the focus of this article.

### 1.2 The approach of a utilization-focused evaluation according to Patton

A utilization-focused evaluation is to be understood as a client-oriented evaluation or actor-oriented evaluation: “*Utilization-focused Evaluation is a process of creatively and flexibly interacting with intended evaluation users about their information needs and alternative methodological options, taking into account the decision context in which an evaluation is undertaken”* [(2), p. 175]. At the core of utilization-focused evaluation is the question of how the results can be concretely used by the individuals for whom the evaluation is being conducted. These so-called intended users are expected to apply the evaluation findings in their practical work, decision-making processes, or program development. Compared to more traditional, summative forms of evaluation—which often focus on retrospective judgments and external accountability—utilization-focused evaluation proves more effective in dynamic and practice-oriented fields such as education, social work, or sport. In these contexts, where continuous development and adaptive learning are essential, the formative, flexible, and stakeholder-engaged nature of utilization-focused evaluation offers clear advantages. The constant and ongoing exchange about the evaluation process and (interim) results with the actors in the field under investigation is the most central element of evaluation. Therefore, the evaluation is designed as a communicative negotiation process between researchers and users. As a result, the process is very personal and situation dependent. For the researchers, this means that they enter into a commitment with the users through the evaluation in order to support them in clarifying the question of what kind of evaluation they need. Patton's approach is criticized with regard to the question of the more precise definition of users. This often arises from the assumption that evaluation-related changes in the research field could only be negotiable with decision-makers on the user side. The result is that usually only a specific subgroup of users could be relevant for the evaluation approach (23).

Qualitative methods are considered particularly suitable for conducting a utilization-focused evaluation (24). This also applies to the evaluation of the BAT Sailing Team presented here. The insights gained and phenomena identified in the surveys cannot be translated into clearly defined and determinable variables or measured. This was also not the aim of the approach. The researchers wanted to reflect on the experiences made together with the users openly and in a communicative process. This pursued the goal of discovering and systematically reconstructing the topics and situations relevant to the participants. The fundamental questions that continue to develop during the research process should also be negotiated between researchers and users on an equal footing. This was done in the sense of a responsive approach that incorporates the reactions of those being studied.

The specific survey and evaluation methods are not predetermined from the outset in a user-focused evaluation. They are selected based on the research object and field of research. Interviews are often selected, as in the case described (24). The researchers opted for guided and episodic interviews (25, 26). The evaluation was based on a pragmatic use (27, 28) of the strategies and (coding) procedures of “grounded theory” (29). This approach is established in qualitative research and is frequently used for evaluation processes (24). The practical research procedure is described in central steps, which must, however, be adapted to the subject area and the specific project. Patton has summarized the important topics in a checklist [(4); see Table 1]:

**Table 1:** Utilization-focused evaluation (U-FE) checklist (4).

Step 1	Assess and build program and organizational readiness for utilization-focused evaluation.
Step 2	Assess and enhance evaluator readiness and competence to undertake a utilization-focused evaluation.
Step 3	Identify, organize, and engage primary intended users.
Step 4	Conduct situation analysis with primary intended users.
Step 5	Identify primary intended uses by establishing the evaluation's priority purposes.
Step 6	Consider and build in process uses if appropriate.
Step 7	Focus priority evaluation questions.
Step 8	Check that fundamental areas for evaluation inquiry are being adequately addressed.
Step 9	Determine what intervention model or theory of change is being evaluated.
Step 10	Negotiate appropriate methods to generate credible findings and support intended use by intended users.
Step 11	Make sure intended users understand potential controversies about methods and their implications.
Step 12	Simulate use of findings.
Step 13	Gather data with ongoing attention to use.
Step 14	Organize and present the data for use by primary intended users.
Step 15	Prepare an evaluation report to facilitate use and disseminate significant findings to expand influence.
Step 16	Follow up with primary intended users to facilitate and enhance use.
Step 17	Metaevaluation of use: Be accountable, learn, and improve.

The researchers moderate the evaluation. The users must be involved in such a way that they are very likely to use the results of the evaluations. This requires that they understand the evaluation process and the results and that they take ownership of the process. Since evaluation cannot be free of values, an essential aspect is that the users and their values, with which they identify, are actively involved in the evaluation process. Only through this active involvement is it possible for users to understand and comprehend the process and the results.

## 2 Utilization-focused evaluation of the BAT sailing team

### 2.1 Starting point of the utilization-focused evaluation

The inclusive BAT Sailing Team 2020 was founded with the aim of breaking down the previous practice of separating sailing and paralympic sailing. The origin of this initiative was a sailing workshop for blind and visually impaired people. The name, which is derived from the word “bat” and alludes to the symbolic transfer: cannot see (well) but can fly (or sail). The founding crew consisted of 3 blind athletes and 4 sighted athletes, with one sighted person using a wheelchair. The founding crew also included 2 organizers and a coach. In 2024, the crew had grown to 13 actively sailing people, 4 of whom are blind, 2 of whom are severely visually impaired and one of whom is deaf. There is also a land team consisting of up to 4 people.^1^ All named impairments are congenital. In Germany, a person is legally classified as blind if they have a visual acuity (visual acuity) of no more than 0.02 (1/50) in their better eye. The athletes with visual impairments mentioned above have visual acuity between 0.05 and 0.3 in their better eye, which is classified as severe visual impairment in Germany.

As soon as the team was founded, it was clear to them that participating in Germany's largest sailing regatta, Kiel Week, would be the highlight of 2021. The J/70 was chosen as a boat class that is not explicitly known for adaptive sailing or has special provisions in terms of accessibility. The aim was to compete with non-(explicitly) inclusive teams in the regular competition in order to make the supposedly exclusive character inclusive from within. It was also clear that this was not to be a one-off project, but merely the start of a long-term initiative for inclusive sailing. One of the team organizers approached the scientists with the idea that professional and sustainable development should be objectively supported by an external body in the best possible way. Individuals from local sports associations, who were aware of the authors' scientific focus from previous joint projects, put them in touch.

In the first meetings between scientists and those responsible for the BAT Sailing team, the possible design of the accompanying evaluation research was discussed together. During this phase, the scientists were given the task of determining the initiative's readiness for evaluation (Step 1 of the checklist) and explaining the processes and purpose of a utilization-focused evaluation. It was emphasized by the scientists in this phase that the evaluation should have a direct utilization for the BAT Sailing team. The scientists also analyzed that the level of development of the BAT Sailing team made an evaluation appear sensible. The two parties agreed on an intensive exchange in the form of annual workshops. To this end, scientists were to observe the BAT Sailing Team's training and competitions and exchange ideas with the athletes. For this purpose, the BAT Sailing team assured the scientists access to the field. This also included arranging interview partners. The BAT Sailing team's willingness to evaluate was rated as high. The field of research itself was considered sensitive by the researchers, as people with and without disabilities interact together and are certainly aware of their special situation. It was assumed that not all participants would have a positive attitude towards the evaluation. Accordingly, it was also expected that not all participants would be willing to be interviewed.

Special consideration was given to the situation of interviewing people with disabilities. The qualifications and experience of the evaluators were analyzed accordingly (Step 2 of the checklist). The team of researchers had the relevant expertise and previous experience from previous fieldwork in the context of inclusive sailing. Likewise, several members of the research group are considered experts in the research field of “inclusion in sports” and have experience in collecting qualitative data in this context (30). Accordingly, the research group engaged in intensive discussions about the upcoming fieldwork and anticipated the possible course of events.

**Figure 1:**
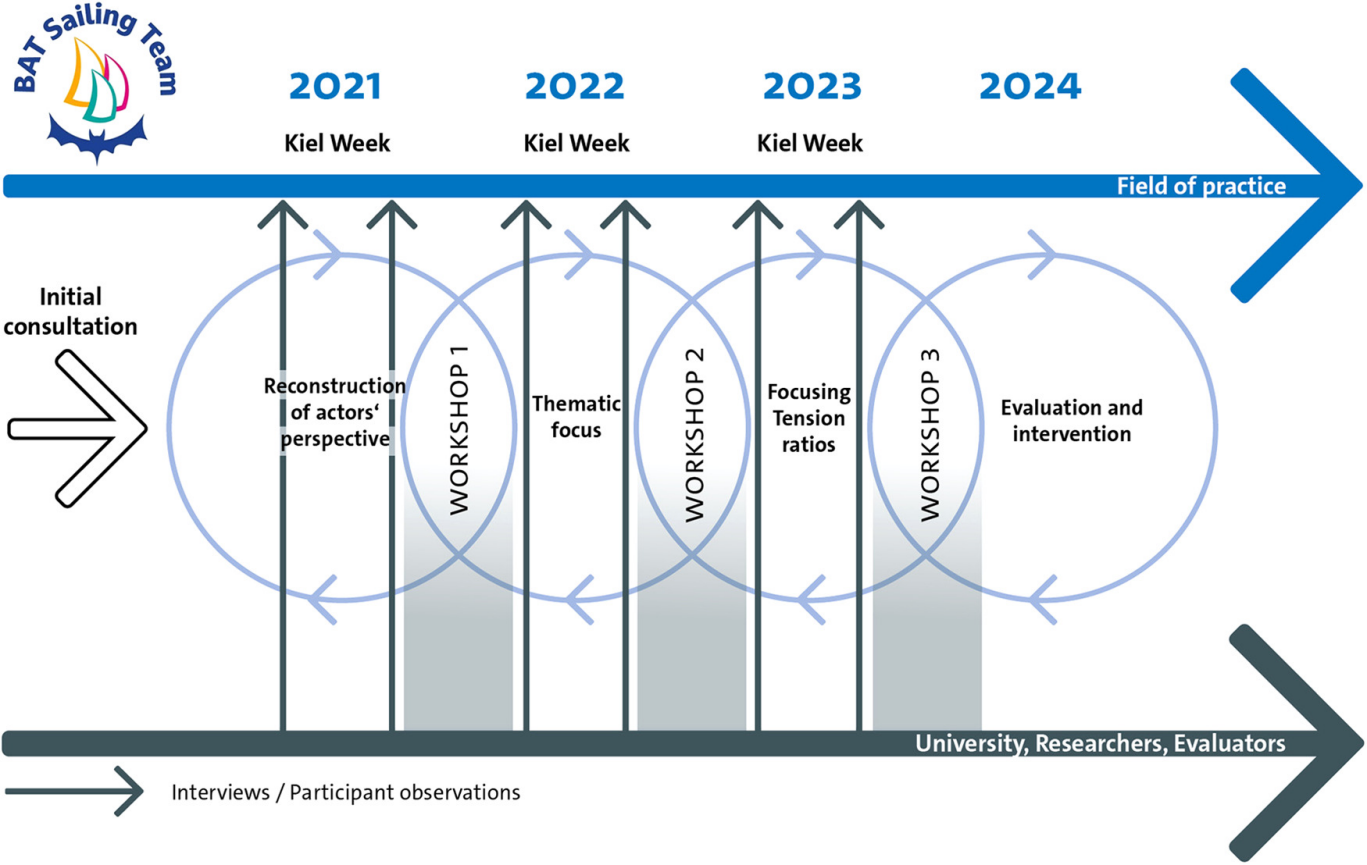
Illustration of the scientific monitoring of the BAT sailing team in terms of a utilization-focused evaluation.

The athletes and those responsible for the BAT Sailing team were identified as the primary beneficiaries of the evaluation findings (Step 3 of the checklist). The researchers had already anticipated this in the first situation analysis, which took place before the kick-off meeting (Step 4 of the checklist). To this end, the homepage and press releases on the BAT Sailing Team were analyzed in detail. In addition, the researchers already had information about the BAT Sailing Team due to previous field access at an inclusive regatta. Contacts had already been made with athletes and information collected. The assumption of the primary utilization for the BAT Sailing Team was confirmed in the initial discussions (see Figure 1).

### 2.2 First year of the evaluation in 2021 – reconstruction of actors' perspectives

The analysis of the situation (Step 4 of the checklist) of the BAT Sailing Team is an ongoing process that runs through the entire evaluation process. In order to harmonize the evaluation process between evaluators and those responsible for the BAT Sailing Team, and thus between scientists and sports practice, several points of contact were initiated between these two status groups. The evaluators observed training sessions and various competitions of the team and also took part in other activities at the sailing club in order to get to know the environment, the training and competition venues, and of course in particular the people involved. These steps were fundamental to the evaluation process, as the trust of those involved in the BAT Sailing team had to be gained. This is due to the form of the evaluation, as in addition to participant observation, interviews with the participants were also part of the evaluation process. The importance of conducting interviews and obtaining the perspectives of the stakeholders emerged from the initial discussions between the researchers and those responsible for the BAT Sailing team and from the observations. It quickly became clear that there were many different perspectives on sailing and the upcoming competitions at Kiel Week. To design a profitable evaluation, these perspectives, many of which were not clear to all participants, had to be identified and systematically analyzed. A qualitative approach was chosen for this, using interviews. To this end, the stakeholders were divided into different groups at an analytical level (athletes with and without disabilities, coaches, organizers). Actors from the groups were interviewed before and after the Kiel Week to be able to reconstruct their perspectives in a comparative manner (see Table 2). Care was taken to include participants from all stakeholder groups to obtain as comprehensive a picture of the situation as possible.

**Table 2:** Overview of interviews and interviewees from 2021.

Name	Role	Sex	Age	Degree of VI	Pre-interview	Post-interview
Alf	Athlete	M	37	Blind	Yes	Yes
Fred	Athlete	M	27	Blind	Yes	Yes
Peter	Athlete	M	30	Blind	Yes	Yes
Lee	Athlete	M	32	Sighted	Yes	Yes
Micheal	Athlete	M	29	Sighted	Yes	Yes
Christa	Athlete	F	53	Sighted	No	Yes
Anna	Coach	F	48	Sighted	Yes	Yes
Nathalie	Organizer	F	37	Sighted/wheelchairuser	No	Yes
Stefan	Organizer	M	52	Sighted	Yes	No

The technique of episodic interviews was chosen, as these aim to present experiences in a general, comparative form. Concrete situations are also reconstructed, and the advantages of narrative and guided interviews are combined (31). Interview guidelines were used, but the interviewers were able to react spontaneously to statements and the individual interview processes and explore both specific attitudes (e.g., to their attitude towards people with disabilities, to competition and result orientation in sport) and concrete episodes from training sessions or competition situations. The interview guidelines therefore contained components that were relatively identical for all stakeholder groups. All interviewees were asked about their individual views on the BAT Sailing Team and the practices that take place there. The focus was also on participation in Kiel Week and the associated expectations (of themselves, the team, the event). There were specific sections for all groups of participants. For example, the athletes were asked about their sporting or competitive biographies and the extent to which these are linked to their sporting expectations of competition in sailing. The organizers and coaches, on the other hand, were asked about their experiences and interpretations regarding the integration of the inclusive team into the existing structures of sailing. This concerned, for example, the anchoring of training times in clubs that had previously worked (less) inclusively, but also the organization of participation in competitive regattas and finally the big event of Kiel Week. The length of the interviews was 38–119 min.

The choice of an explorative, qualitative design is also justified by the desire of those people in charge in the BAT Sailing team to map action processes as accurately as possible and to be able to influence them directly with the help of the results. In the data collection (points 9, 10 and 13 of the checklist), the premise was thus taken into account that the data collection should be carried out with constant attention to the question of utilization [cf. (30)]. Careful management of the evaluation process was ensured through ongoing reflection on the evaluation process by the researchers. The researchers were also in constant communication with those responsible in the BAT Sailing team. Rules were agreed for field access, as this is a very sensitive field. In addition, all evaluators were familiar with the context of disability sport and had previous experience in research in this field (32). The job shadowing sessions made it possible to identify various needs of the stakeholders. All members of the BAT Sailing team were also made aware that the evaluation should have a direct utilization for the team.

The direct confrontation of the actors with the data and the results of the analysis (Step 14 of the checklist) took place in a workshop held six months after Kiel Week. By the time of the first workshop, 15 interview transcripts and numerous field notes had been produced (33). The data was analyzed using the coding methods of Grounded Theory (29). The results were discussed in research team meetings. Open and axial coding were primarily used for the analysis. Selective coding was not used, as open and axial coding were sufficient for the thematic analysis. The character of the utilization-focused evaluation became apparent in the evaluation processes. After the initial open coding processes, the phenomena that (provisionally) appeared to be relevant were discussed with those responsible in the BAT Sailing team. The interview guidelines were adapted on the basis of these discussions. Ongoing data collection and axial coding allowed the categories identified to be substantiated and further differentiated. In addition, a triangulation of perspectives (34) of the different stakeholder groups took place. This allowed the different perspectives of the stakeholder groups to be contrasted.

When analyzing the data, it emerged that the perspectives of the stakeholders had to be analyzed on the three levels of “event”, “group” and “individual”. At the “event” level, the tension between “participation for all vs. professionalization” appeared relevant. At the “group” level, the continuum “develop - apply ‘new’ communication - pass on” could be presented. At the “individual” level, the participants' statements were illustrated with the area of tension “Between childhood dream and new, enjoyable hobby”. At the workshop, the previous steps in the field and the results of the data analysis to date were presented, discussed and reflected upon. The workshop thus served to continuously identify the primary intended utilizations, to focus the evaluation and to concretize the evaluation plan (steps 4, 5, 6, 7 and 8 of the checklist).

In order to achieve the greatest possible utilization for the optimization of processes at the BAT Sailing team (Step 5 of the checklist), a preliminary meeting was held between scientists and those people responsible in the BAT Sailing team to plan the workshop. A corresponding strategy for the workshop was developed here. It was decided that the phenomena should be discussed in small groups using interview quotes. In addition, the scientists were to present their analytical steps so that everyone could understand the necessity of the evaluation. The scientists were to take on a moderating role in the workshop. In the workshop, the researchers aimed to prepare the data in such a way that it was understandable and relevant for the primary intended users (Step 14 of the checklist). This also means, for example, that the workshop processes and visual content were always verbalized to ensure greater accessibility. Attention was also paid to interactive breaks in the presentation, during which questions could be clarified at any time. The participants were confronted with interview quotations and thus with authentic material [in the sense of “face validity”; (35), p. 93] and thus encouraged to critically discuss their practice.

The workshop was organized for a time frame of three hours. A welcome address was followed by a short overview presentation on organization and structure and on the purpose of the type and manner of evaluation. The presentation led into group discussions (36). Thematic tables were prepared for this purpose, which were derived from the data analysis.

The first table was entitled: >Childhood dream vs. nice hobby - How to deal with individual demands in a group?<. The participants should discuss the following questions: (A) What does sailing and specifically taking part in Kiel Week mean to you? (B) How should the group's requirements be developed?

The second table was entitled: >Team growth between participation for all and professionalization<. The participants should discuss the following questions: (C) Does the team need to grow? (D) What are the challenges in terms of team growth?

With the help of impulses from short interview quotes, moderated discussions followed under the key questions described. The participants were also asked to develop alternative courses of action for the future and define concrete steps. The discussions and results of the workshop formed the basis for the further course of the evaluation. The researchers left it up to the BAT-Sailling team to decide whether the evaluation should continue.

### 2.3 Second year of the evaluation 2022 – thematic focus

Those people responsible for the BAT Sailing team contacted the scientists after the workshop and reported on the decision to also be evaluated as part of Kiel Week 2022. They reported that the team had decided to tackle the questions identified in the first workshop: “Childhood dream vs. nice hobby - how to deal with individual demands in the group?” and “Team growth between participation for all and professionalization?”. This meant that the people in charge wanted to continue to grow. The aim was to start not just with one, but with two boats at Kiel Week. The respective crews of the boats were also to be arranged in such a way that a corresponding increase in performance would also be possible. To achieve this, new athletes should be acquired. Ambitious and experienced athletes should also be given the opportunity to train and compete with a focus on success. This also means that communication on and off the boat between people with and without disabilities should be optimized.

Based on these aims of the BAT Sailing team, the scientists developed the procedure for the further evaluation. Interviews were again conducted before and after Kiel Week and training sessions were also observed. Data collection focused on the questions and problems described above. To this end, the guidelines for the interviews were redesigned accordingly and the aforementioned topics were central to the questions, e. g. “How is it that you are now sailing in boat number 1/2?” or “How do you rate the ambitions of your crew?” Ten interviews with eleven people were conducted before and also after Kiel Week (see Table 3).

**Table 3:** Overview of interviews and interviewees from 2022.

Name	Role	Boat	Sex	Age	Degree of VI	Pre-interview	Post-interview
Alf	Athlete	1	M	38	Blind	Yes	Yes
Fred	Athlete	2	M	28	Blind	Yes	Yes
Peter	Athlete	1	M	31	Blind	Yes	Yes
Lee	Athlete	1	M	33	Sighted	Yes	Yes
Micheal	Athlete	2	M	30	Sighted	Yes	Yes
Christa	Athlete	2	F	54	Sighted	No	Yes
Jasmine	Athlete	1	F	26	Sighted	Yes	Yes
Cathy	Athlete	2	F	31	Sighted/deaf	Yes	No
Marla	Athlete	2	F	29	Sighted	Yes	Yes
Manu	Athlete	2	F	32	Visually impaired	Yes	Yes
Anna	Coach	-	F	49	Sighted	Yes	Yes

In the second year described above, the evaluation was therefore essentially focused (Step 7 of the checklist), although points 4, 5 and 6 were also revisited and reflected upon.

In the workshop, the participants worked in small groups. They were asked to develop guiding principles for joint action in the BAT Sailing team and present them to each other. The idea of developing guiding principles arose in a preliminary exchange between the researchers and the team organizers. The scientists had already found this process beneficial in a previous benefit-focused evaluation in the context of inclusive handball and the organizers of BAT sailing expressed the desire for a concrete proposal from the scientists so that the team members would have a point of reference for orientation for individual adaptation. The groups of four people each worked according to the think-pair-share principle (T-P-S). The “mission” proposed by the scientists was: “We sail (Kiel Week) in a performance-oriented AND inclusive manner”. The following sentence was proposed by the scientists as a guiding principle: “In order for us to realize the mission with fun, everyone should have sailing skills or be able to acquire them quickly”.

In the T-P-S, the participants were first asked to read, correct and expand the university suggestions on their own (Think). They then shared, compared and discussed their own suggestions with another person (Pair). This was then presented to the small group (Share). The guiding principles were then discussed by the entire group of participants (plenary). First of all, it should be noted that almost all athletes asked for the time available to work on the first step to be doubled. The original 15 min thus became approx. 30 min. The subsequent discussion clarified the reasons for the need: the fact that the team was sailing in two different high-performance boats for the first time obviously led to new group-finding processes within the BAT Sailing Team, which also led to tensions and friction between athletes from the different boat teams over the course of the season. While the above-mentioned formulation of the mission was felt to be adequate by all team members, opinions were divided on the proposed guiding principle. Those athletes who felt they belonged to the less performance-oriented boat rejected the sentence in this form and formulated it as “In order for us to implement the mission with fun, we need good communication”. In a discussion, this was accepted by all athletes as a common guiding principle and was described as essential, especially considering the addition of a deaf athlete to the team.

### 2.4 Third year of the evaluation 2023 – focusing tension ratios

The BAT Sailing team also decided to continue the scientific evaluation in 2023. The focus was also on Kiel Week. In the second workshop, which took place six months before Kiel Week 2023, the obvious tension ratios that have accompanied and shaped the team since its participation in Kiel Week were once again highlighted. The question of the further growth of the team and possible professionalization were central to this. In order to analyze this further, interviews were again conducted with the members before and after Kiel Week (see Table 4).

**Table 4:** Overview of interviews and interviewees from 2023.

Name	Role	Boat	Sex	Age	Degree of VI	Pre-interview	Post-interview
Alf	Athlete	1	M	39	Blind	Yes	Yes
Fred	Athlete	2	M	29	Blind	Yes	Yes
Peter	Athlete	1	M	32	Blind	Yes	Yes
Lee	Athlete	1	M	34	Sighted	Yes	Yes
Micheal	Athlete	2	M	31	Sighted	Yes	Yes
Christa	Athlete	2	F	55	Sighted	No	Yes
Jasmine	Athlete	1	F	27	Sighted	Yes	Yes
Marla	Athlete	2	F	30	Sighted	Yes	Yes
Manu	Athlete	2	F	33	Visually impaired	Yes	No
Stefan	Organizer	-	M	54	Sighted	No	Yes

The third loop focused on the “promotion of utilizations” (point 16 of the checklist). Building on the knowledge gained up to this point, the question was how the actions at BAT Sailing could be improved in concrete terms. Particular consideration was given to reconciling the various interests of the members. To this end, the creation of an organizational chart was suggested. In the workshop, which was again held a few months after Kiel Week, work was again carried out in small groups of four people using the think-pair-share principle. This had proved successful in the previous workshop and was also requested by the participants. The guiding principles developed in the last workshop were used for this. These were to be reconsidered in light of the experiences of the previous Kiel Week. The group then had the task of creating an organizational chart for the further internal team organization. The scientists had put forward the idea for an organization chart in the preliminary discussion for the workshop with those responsible for the BAT Sailing team. The organization chart was intended to clarify the structure of the team and define and define responsibilities. The reason for this was the fact that, despite the team being divided into two boats with different goals, some team members had to use these structures flexibly for the purpose of helping out. In addition, there are tasks outside of active sailing, such as public relations work, which are carried out for the entire initiative regardless of the respective boat. Those responsible welcomed this idea for the organization of the workshop. However, in the discussion at the workshop itself, the participating athletes agreed that they would not need an organizational chart. This decision was preceded by a process of exchange that focused on the situation of two boats and their respective crews. The athletes came to the realization that they see themselves as one big team, regardless of any division into performance-oriented and participation-oriented. In connection with this, areas of responsibility outside of sailing, such as public relations and acquiring sponsors, were also formulated as areas that are fundamentally open to everyone.

## 3 Communication on and off the boat as reflected in the utilization-focused evaluation

The interviews were transcribed and subsequently analyzed in line with the pragmatic application of coding procedures from Grounded Theory (27, 28). By combining inductive and deductive analytical steps, underlying structures within the data material were revealed. The following section draws on anchor quotes from the interviews to illustrate how communication within the BAT Sailing team has evolved over the course of three years. These anchor quotes represent particularly salient excerpts that highlight relevant phenomena and shed light on how members of the BAT Sailing team have responded to and dealt with them. The focus here is on the situation between sighted and blind athletes in particular. The interviews in the first year of the utilization-focused evaluation were characterized, among other things, by the fact that sighted athletes had to develop and learn many new components in communication. This is illustrated by the following example

Lee (2021 – first year): “What we've all learned - me in particular - is communication. I never go on a boat anymore without saying: “I'm getting on the boat.” That we have learned to always communicate everything, briefly and concisely, with simple sentences that are familiar. I've already transferred that to all the boats I sail on.”

It is interesting to note at this point that the sighted athlete not only uses this type of communication on the BAT boat, but also on other boats in regular (i.e., non-inclusive) sailing. This indicates a positive interpretation of these “new” communication practices, which are obviously seen as generally useful for sailing, as other interview passages also show. Many athletes without disabilities reflect on their own behavior and their previous communication in the context of sailing. This is stimulated by the experience of communication in the BAT Sailing Team. The resulting “new” communication is perceived as enriching, which is also shown by another quote from a female sighted athlete:

Christa (2021 – first year): “We all saw ourselves as very equal.. where everyone has their own needs. For most people, communication was very important, talking about everything.. especially because of the blind people involved. And I think the fact that we took the time to do this meant that we were really well coordinated, that our processes were really smooth if we always discussed everything in detail before and after training. And then I also realized that you don't do it that way with sighted people.. there's much more non-verbal.. just by seeing what the other person is doing, I know what they're up to. (..) What I'm getting at is that it helped us a lot to verbalize everything and work on our communication.. which maybe other teams don't do. (..) I think that was a great learning gift.”

The detailed verbalization obviously also leads to the atheletes' thinking about how blind people experience the processes. This change of perspective can be seen as a beneficial learning process that goes beyond sailing (or sports). This is an important insight, especially for people without disabilities. In addition to the perspectives of sighted athletes described so far, the participating blind athletes also describe enriching processes. It is interesting to note that these are also located in the context of communication among the sighted athletes.

Alf (2021 – first year): “Our sighted people also learn from us - communication, for example - they talk differently. Non-verbal communication only with tactile possibilities - otherwise not possible with the blind. That's why they have to learn to talk more - but not endlessly. (..) We have developed certain commands.”

The blind athlete mentions learning from the sighted athletes. He also describes that a pragmatic procedure was developed for this process, which was adapted to the needs of sailing. In this quote, a communicative distinction is also made between the “sighted” and “blind” groups. This is also evident elsewhere in the interviews:

Jasmin (2022 – second year): “It's really enriching for me, because they bring in completely new ideas that you don't really have on your radar as a sighted athlete. They do a lot of listening, they observe the sounds that they hear around them, the sounds that their own boat makes. They feel a lot. That's really enriching and really cool input. And for me, it's also an impetus to rethink how I've sailed as part of a team so far. (..) A lot is about non-verbal communication - and I find myself doing that again and again. When I go on a boat with blind people, it just doesn't work.”

In this quote, here from a sighted athlete, a distinction is also made between the in-group (we, the sighted) and the out-group (the others, the blind). The language manifests this distinction. The evaluators were already familiar with this distinction from other contexts [e.g., inclusive handball; (30, 37)].

In the second year of the evaluation, communication was discussed less in the interviews. Obviously, the learning effect or the effect of being confronted with something new and unfamiliar gave way to a certain routine, which was also described:

Lee (2022 – second year): “So we always say windward or leeward and that also helps us the other way round in the team, so that everyone knows exactly what's going on.”

The sighted athlete describes a sail-specific command that is effective for everyone. This also seems to economize the work in the team. Another example relates to a different level of interpersonal communication:

Lee (2022 – second year): “I think what's important is simply that we've developed as a team. Especially with Cathy, she's really happy that we're learning the signs and that we also like to learn some nonsense signs that hardly anyone actually needs.”

The sighted athlete describes the feelings of a sighted and deaf athlete, who is obviously pleased that she is not only learning sailing-specific commands as signs and the usual tone of voice, but also colloquial language and “trash”. In general, it became clear in the first two years that communication between sighted and blind athletes is very important and that Cathy as a new team member has brought further development. This was also evident in the interviews in the third year, as this excerpt shows:

Michael (2023 – third year): “Boat 1 [the performance-oriented one of the two boats] was simply better in sporting terms. That's the team that is better coordinated and also has better communication with each other. In my opinion, this is also because they have a different communication culture and not so many obstacles to communication. In other words, they consciously take the time to talk to each other before they arrive at the camp. So when they came off the boat, they deliberately met briefly outside the camp, talked to each other about the day, what each individual was allowed to say, without interrupting the other, but they always have their rhythm, that they go through the boat from, I think, the front to the back, i.e., the positions in which they sit on board. In this order, they talk about what they found good, bad, moving, whatever, without interrupting or judging the other person. They talk about their own perceptions. And when they've finished, they talk about what needs to change.”

The sighted athlete, who sailed in the second boat of the BAT Sailing Team during Kiel Week, describes here that the team of Boat 1 was more successful in sporting terms. He attributes this to increased clarity of communication within the team. This clarity in communication between the blind and sighted athletes was also achieved by the team setting up and ritualizing appropriate communication situations away from the sailing situation. This resulted in a higher level of commitment. This was practiced and led to sporting success, which was also visible. This culture of communication provided security and self-confidence and demonstrates the positive cooperation between athletes with and without disabilities. There was a clearer distribution of responsibilities and tasks, which led to better processes.

## 4 Discussion

### 4.1 Focusing the methodological approach

The presentation and reflection of the BAT Sailing project shows the potentials and limitations of the research methodological approach in the context of utilization-focused evaluation in a specific setting. By presenting selected evaluation results to the BAT-Sailing team, the scientists reflected on their own behaviors and used the workshops to critically deal with them. As shown, this led to an awareness of the many different needs and interests which were dealt with positively by the participants. At this point, it seems important to mention that the scientists perceived the close and personal exchange as always open-minded and cordial and felt that they were accepted by the team as part of the self-critical development that had been desired from the outset. However, this also meant that the researchers had to constantly reassure themselves of their role and, for example, did not take the lead in discussions during the workshops, which could have contradicted the actual user orientation. The scientists found it particularly interesting that Cathy, a deaf athlete, joined the team in the second year, but this did not lead to any further discussion of the topic (more on this below). The evaluation thus had a direct influence on practice and did not remain without consequences in the evaluators' sphere of knowledge (38), on the criticism of this phenomenon, which frequently occurs in evaluation practice). This process was made possible by the mutual commitment of researchers and actors from the BAT Sailing team to work together. The flexible structure of the evaluation was also beneficial here, allowing the needs of those being evaluated to be addressed. This was reflected, among other things, in the flexibility in the choice of topics for the workshops or the adjustments that were made during the work phases in the workshops. This was always geared towards the needs of the participants. In addition to the general approach in the evaluation, this shows a great openness to systematically address relevant topics of the users and thus the strong orientation towards the usefulness for the users or stakeholders (as those primarily affected by the results), which is an important quality criterion of utilization-focused evaluation (35). With regard to feasibility as a further quality criterion of utilization-focused evaluation (35), it can be stated that all processes of data collection and presentation met with the acceptance of the BAT-Sailing stakeholders. In addition, the evaluation met ethical standards, and the evaluators showed great consideration for the particularities of the field being researched. The quality criterion of correctness (35) was therefore met. In addition, the data collection and evaluation instruments used correspond to the quality criteria of empirical social research, which means that the quality criterion of accuracy (35) was fulfilled. The checklist of utilization-focused evaluation represents a suitable orientation framework for the implementation of the evaluation. However, adjustments or omissions were also made to components, for example, the extensive concrete simulation of utilizations (Step 12 of the checklist) was omitted. Although the possible consequences of a concrete approach were repeatedly pointed out in advance in the discussions, no concrete work was carried out on fabricated hypothetical data and thus on possible, potential results of future surveys. This procedure once again shows the importance of trusting cooperation between both sides in such a form of evaluation. To build up such a relationship of trust, the researchers also need to know the respective characteristics and customs of the field as extensively and precisely as possible, which usually includes corresponding internal information and processes. Through previous experience from other areas of sport on the way to inclusive structures, the researchers were able to make transfers to sailing and, above all, anticipate barriers that the inclusive team encountered in interaction with their performance-oriented sailing environment. Due to the open exchange about this in the context of the interviews, but also apart from this, a corresponding relationship of trust was built up between the athletes and the researchers over the years. This bond cannot be taken for granted when initiating a similar project. In the project presented here, for example, it was only after several years that concrete interventions began to be carried out in practice, as this requires a great deal of trust on the part of the actors in the field. In general, the maxim that users have a right of veto on all ideas of the researchers was followed. Therefore, all research ideas and interests of the researchers were discussed with those responsible for the initiative and modified if necessary. This openness restricted the researchers in some places, but was unavoidable in the interests of the project and in retrospect can be seen as a strength of the project (23).

### 4.2 Focus on selected results

The findings on phenomena like “participation for all vs. professionalization” and “develop - apply “new’ communication - pass on” presented here must also be viewed in this light, namely as those phenomena that the BAT Sailing team considers relevant for their own practice and its further development. In conclusion, it can be said that the BAT Sailing Team has repeatedly committed itself to the goal of practicing inclusive and competitive sailing in recent years, while at the same time ensuring the highest possible level of participation for all (new) team members. The tensions and frictions that have arisen in the process are outlined above, along with possible solutions, such as the interest-based division into two boats with different aspirations for competitive success. Another solution, or rather a prerequisite for successful joint action, is the new communication. Although these are supposed peculiarities of communication between primarily sighted and blind people in the context of (performance-oriented) sailing, the athletes' descriptions suggest that these peculiarities can be profitably transferred to other (sporting) contexts. This is perhaps an area of strength in the context of inclusive sailing for sighted and visually impaired people, from which other inclusion-oriented areas of sport could benefit and which should be given more attention by sports scientists. In any case, it should be noted that in the context of the present utilization-focused evaluation, the phenomenon of “new” communication was attributed a fundamental benefit for all participants, but especially – from the athletes' point of view – those athletes without visual impairment benefited from the jointly developed communication. Compared to previous research findings, this benefit of inclusive sailing can therefore be attributed to the side of people without disabilities. To what extent exactly the types of communication developed further when a sighted and deaf athlete, Cathy, joined the team, remains largely open in this article. The main reason for this is the strict orientation towards the principles of the utilization-focused evaluation. Although practice may be more strongly influenced by a further development of communication under these conditions, the users (the athletes) do not currently attach excessive importance to this. The authors consider it important to emphasize that this can change and thus become the focus of further utilization-focused evaluation.

## 5 Conclusion

Research in the field of competition-oriented or competitive and at the same time inclusive sports remains rare. This should also be noted in the context of sailing. With regard to sailing together by sighted and blind or visually impaired people, this article has primarily achieved two things: Firstly, by providing detailed presentation and reflection of a utilization-focused evaluation, concrete possibilities for a qualitative, exploratory approach in a field that has been little researched have been offered. This approach requires time and mutual commitment – from both practitioners and researchers – but with a flexible and user-oriented design, it is a promising approach for critical further development, also for both sides. Second, the article provides concrete results regarding the supposedly necessary specific communication in such an inclusive team constellation. Other research contributions also attach particular importance to communication (14, 22). However, this article highlights that all athletes benefit from the communication developed jointly, but that athletes without impairments in particular find the transfer of these learnings to other areas (of sport) beneficial. Future research on inclusive and competitive sports should explore these benefits further.

## Data Availability

The original contributions presented in the study are included in the article/Supplementary Material, further inquiries can be directed to the corresponding author.

## Footnotes

^1^Further information on the BAT Sailing Team can be found at: https://www.wir-sind-wir.org/bat-sailing-team/
